# Identification of *qRBS1*, a QTL involved in resistance to bacterial seedling rot in rice

**DOI:** 10.1007/s00122-013-2145-2

**Published:** 2013-06-25

**Authors:** R. Mizobuchi, H. Sato, S. Fukuoka, S. Tsushima, T. Imbe, M. Yano

**Affiliations:** 1 National Institute of Agrobiological Sciences, Kannondai 2-1-2, Tsukuba, Ibaraki 305-8602 Japan; 2 National Agriculture and Food Research Organization (NARO), National Institute of Crop Science, Kannondai 2-1-18, Tsukuba, Ibaraki 305-8518 Japan; 3 National Institute of Agro-Environmental Sciences, Kannondai 3-1-3, Ibaraki, 305-8604 Japan; 4 National Agricultural and Food Research Organization, 3-1-1 Kannondai, Tsukuba, Ibaraki 305-8517 Japan

## Abstract

Bacterial seedling rot (BSR), a destructive disease of rice (*Oryza sativa* L.), is caused by the bacterial pathogen *Burkholderia glumae*. To identify QTLs for resistance to BSR, we conducted a QTL analysis using chromosome segment substitution lines (CSSLs) derived from a cross between Nona Bokra (resistant) and Koshihikari (susceptible). Comparison of the levels of BSR in the CSSLs and their recurrent parent, Koshihikari, revealed that a region on chromosome 10 was associated with resistance. Further genetic analyses using an F_5_ population derived from a cross between a resistant CSSL and Koshihikari confirmed that a QTL for BSR resistance was located on the short arm of chromosome 10. The Nona Bokra allele was associated with resistance to BSR. Substitution mapping in the Koshihikari genetic background demonstrated that the QTL, here designated as *qRBS1* (*quantitative trait locus for RESISTANCE TO BACTERIAL SEEDLING ROT 1*), was located in a 393-kb interval (based on the Nipponbare reference genome sequence) defined by simple sequence repeat markers RM24930 and RM24944.

## Introduction

*Burkholderia glumae* causes bacterial seedling rot (BSR) and bacterial grain rot (bacterial panicle blight) in rice (*Oryza sativa* L.), which are increasingly important diseases in global rice production (Ham et al. 2011b). Since disease caused by *B. glumae* was first discovered in Japan (Goto and Ohata 1956; Goto et al. 1987; Kurita and Tabei 1967; Uematsu et al. 1976a), it has also been reported in other countries in East Asia (Azegami 2009; Chien and Chang 1987; Cottyn et al. 1996a; Cottyn et al. 1996b; Jeong et al. 2003; Luo et al. 2007; Trung et al. 1993) and Latin America (Nandakumar et al. 2007b; Zeigler and Alvarez 1989). In the USA, *B. glumae* has been identified as the major causal agent of bacterial grain rot (Nandakumar et al. 2005, 2009; Shahjahan et al. 2000). In the southern USA, yield losses caused by outbreaks of bacterial grain rot in rice fields in Louisiana were as much as 40 % in 1995 and 1998; significant losses caused by this disease were also experienced in more recent years (Ham et al. 2011a, b; Nandakumar et al. 2009; Shahjahan et al. 2000; Zhou et al. 2011). Because the optimal temperature range for the growth of *B. glumae* is relatively high (30–35 °C) (Kurita et al. 1964; Tsushima et al. 1986), this pathogen has emerged primarily in tropical and semi-tropical countries. Global warming may enable disease caused by *B. glumae* to reach destructive levels (Ham et al. 2011b); thus, this pathogen should be recognized as a potential threat to the world’s rice production (Ham et al. 2010).

Seeds contaminated with *B. glumae* are sown and transplanted into fields and in some cases BSR appears (Azegami 2009; Azegami et al. 1988; Ham et al. 2011a; Tsushima 1996; Tsushima et al. 1989, 1991, 1996). Occasionally, the typical symptoms do not appear and plants with infected leaf sheaths seem to grow normally, making it difficult for farmers to identify the need to apply preventive agricultural chemicals. However, at heading, panicles are infected by vertical distribution from contaminated leaf sheaths. Thus, infected seeds cause both BSR and grain rot. The infection reduces yield owing to spikelet abortion and infected seeds also cause BSR and grain rot in the next generation (Ham et al. 2011b). Infection of rice seeds with *B. glumae* is associated with several endogenous and exogenous factors such as host susceptibility, inoculum density, humidity and temperature conditions (Azegami 2009; Goto 1983b; Mogi 1984a, b, c). Both high humidity and high temperature are conducive to infection of the seeds (Azegami 2009). In Japan, most rice seeds are sown in nursery boxes, and seedlings are moved to nursery beds before transplanting. Because nursery boxes are maintained under relatively high temperatures (28–30 °C) to promote good germination, BSR tends to occur in nursery boxes if seeds are infected (Uematsu et al. 1976a, b). Seed treatment with oxolinic acid, a quinoline derivative, is a major means for the control of BSR in Japan (Hikichi 1993a, b; Hikichi et al. 1989). However, the occurrence of strains naturally resistant to oxolinic acid has been a serious limitation to this method of disease control (Hikichi et al. 2001; Maeda et al. 2004, 2007). Recently, plant rot after transplanting caused by *B. glumae* also has been reported in Japan (Hasegawa 2012), indicating that BSR has been more widespread than in the past.

Many studies have been performed to understand the genetic control of resistance to bacterial grain rot and several cultivars appear to be resistant to bacterial grain rot (Goto and Watanabe 1975; Groth et al. 2007; Imbe et al. 1986; Mogi and Tsushima 1985; Nandakumar et al. 2007a; Nandakumar and Rush 2008; Pinson et al. 2010; Prabhu and Bedendo 1988; Sayler et al. 2006; Sha et al. 2006; Takita et al. 1988; Wasano and Okuda 1994; Yasunaga et al. 2002). Using resistant cultivars, QTLs for resistance to bacterial grain rot have been reported (Mizobuchi et al. 2013; Pinson et al. 2010). In contrast, few reports about resistance to BSR have been published, because BSR resistance is a complex characteristic influenced by environmental factors (Azegami 2009; Goto 1982; Mogi 1984a, b, c). Although *B. glumae* causes both seedling rot and grain rot, no correlation between the resistance to each was observed (Goto 1983a). To date, no source of complete resistance has been identified (Goto 1983a; Sayler et al. 2006), although some cultivars show partial resistance (Goto et al. 1987; Hirashima and Wakimoto 1983; Sayler et al. 2006). However, the molecular mechanisms for resistance to BSR have not been analyzed and no breeding program for BSR resistance has been established.

In this study, we performed genetic analysis of resistance to BSR and successfully detected a QTL for resistance on chromosome 10 using chromosome segment substitution lines (CSSLs) developed from the cultivars Nona Bokra (resistant) and Koshihikari (susceptible). We also verified the effect of this QTL using an F_5_ population derived from a cross between a resistant CSSL and Koshihikari. We further delimited the candidate genomic region of the QTL by substitution mapping.

## Materials and methods

### Plant materials

To identify the chromosomal regions controlling resistance to BSR, 44 CSSLs, which were previously developed from a cross between Nona Bokra (resistant) and Koshihikari (susceptible) (Takai et al. 2007), were used. Nona Bokra, the donor parent, exhibits extremely late heading under natural field conditions in the summer in Tsukuba, Japan. Therefore, mature seeds of Nona Bokra were obtained using short-day equipment to promote heading.

On the basis of our initial results, we performed additional experiments with SL535, a resistant CSSL in which part of the short arm of chromosome 10 of Koshihikari was substituted with the corresponding segment of Nona Bokra. Forty-six F_2_ plants were produced by crossing of SL535 with Koshihikari, and 46 F_5_ lines were developed by the single-seed-descent method and used for QTL analysis. For substitution mapping of the QTL for BSR, an additional 82 F_2_ seeds were sown in a growth chamber room. Out of the 128 F_2_ plants (46 + 82), we selected 9 with recombination in the short arm of chromosome 10 and obtained F_3_ seeds. From each of the F_3_ lines, we selected an F_3_ plant that was homozygous for the recombinant chromosome and used the F_4_ lines derived from the F_3_ plants for substitution mapping.

### Assessment of BSR resistance

The bacterial strain used in this study was *B. glumae* MAFF 301682 (MAFF: Culture collection of NIAS Genebank, National Institute of Agrobiological Sciences, formerly the culture collection of Ministry of Agriculture, Forestry and Fisheries, Japan), which was virulent on a large number of cultivars and maintained at the National Institute of Vegetable and Tea Science. Bacterial inocula were incubated on LB medium with 2 % agar at 28 °C for 4 days and then adjusted to a concentration of 10^8^ per ml with sterilized, deionized water. Rice seeds were sterilized by soaking in chlorine bleach (available chlorine 2.5 %) for 30 min and rinsed carefully with sterilized water. The sterilized seeds were placed in a freshly prepared bacterial suspension and held under vacuum (0.2 MPa) for 3 min. The inoculated seeds were dried overnight and then soaked in sterilized water for 2 days in a plant growth chamber at 27 °C. The seeds were then sown in a sterilized soil (Bonsol No. 2, Sumitomo Kagaku Kougyo, Osaka, Japan) and incubated in a growth chamber at 27 °C with 80 % humidity under a 14-h photoperiod with a photon flux intensity of 13.5 μmol m^−2^ s^−1^. Disease symptoms were scored 8 days after sowing on a scale of 1–3, where 1 = no symptoms, 2 = sheaths with reddish-brown lesions (mild infection), and 3 = necrotic seedlings or seeds with no germination (severe infection). The BSR ratio was calculated from these scores as:

$${\text{BSR ratio }}\left( \% \right) \, = \, \left( {15 \,-\, \left( {N_{0}-N_{1} /2} \right)} \right) \times 100/15$$

where *N*_0_ is the number of seedlings with score 1, *N*_1_ is the number of seedlings with score 2, and 15 is the number of seeds per replication. There were four replications per inoculation. As a control, we germinated uninoculated seeds and confirmed that the average germination rate was >90 %.

### DNA extraction and simple sequence repeat (SSR) marker analysis

Total DNA was extracted from leaves by the CTAB method (Murray and Thompson 1980). To obtain SSR markers showing polymorphism between Nona Bokra and Koshihikari, SSR motifs were surveyed in the target chromosome regions (IRGSP 2005); the informative SSR markers identified by this screening were then used for genotyping of F_5_ plants. PCR amplifications were performed in 5-μl reaction mixtures containing 1.0 μl (10 ng) DNA, 2.5 μl of KAPA2G Fast ReadyMix (2×) (Kapa Biosystems, Boston, MA, USA), 0.15 μl of a 20-pM mixture of forward and reverse primers (20 pM of each primer type), and 1.35 μl H_2_O. PCR consisted of an initial denaturation for 1 min at 95 °C; 35 cycles of 10 s at 95 °C, 10 s at 55 °C, and 1 s at 72 °C; followed by a final extension for 30 s at 72 °C. PCR products were separated by electrophoresis in 3 % Agarose Type I gel (Sigma-Aldrich, St. Louis, MO, USA) at 150 V for 180 min. 5 μl of 50 ng/μl DNA was used in the SNP analysis. We used a 384-plex set of SNP markers selected from diverse accessions of cultivated Asian rice (Ebana et al. 2010). Genotyping was performed using the GoldenGate BeadArray technology platform (Illumina Inc., San Diego, CA, USA). These SNPs were detected using the Illumina Bead Station 500G system. All experimental procedures for the SNP typing followed the manufacturer’s instructions.

### Statistical and QTL analysis

Linkage mapping was performed using version 3.0 of MAPMAKER/EXP software (Lander et al. 1987), and the Kosambi map function was used to calculate genetic distances. QTL analyses were performed using composite interval mapping, as implemented by the Zmapqtl program (model 6) provided in version 2.5 of the QTL Cartographer software (Wang et al. 2005). Genome-wide threshold values (*α* = 0.05) were used to detect putative QTLs on the basis of the results of 1,000 permutations. The significance of the difference in seedling rot was determined by Dunnett’s test (JMP version 9.0 software, SAS Institute, Cary, NC, USA).

## Results

### Identification of a candidate chromosomal region for resistance to BSR in the CSSLs

**Fig. 1:**
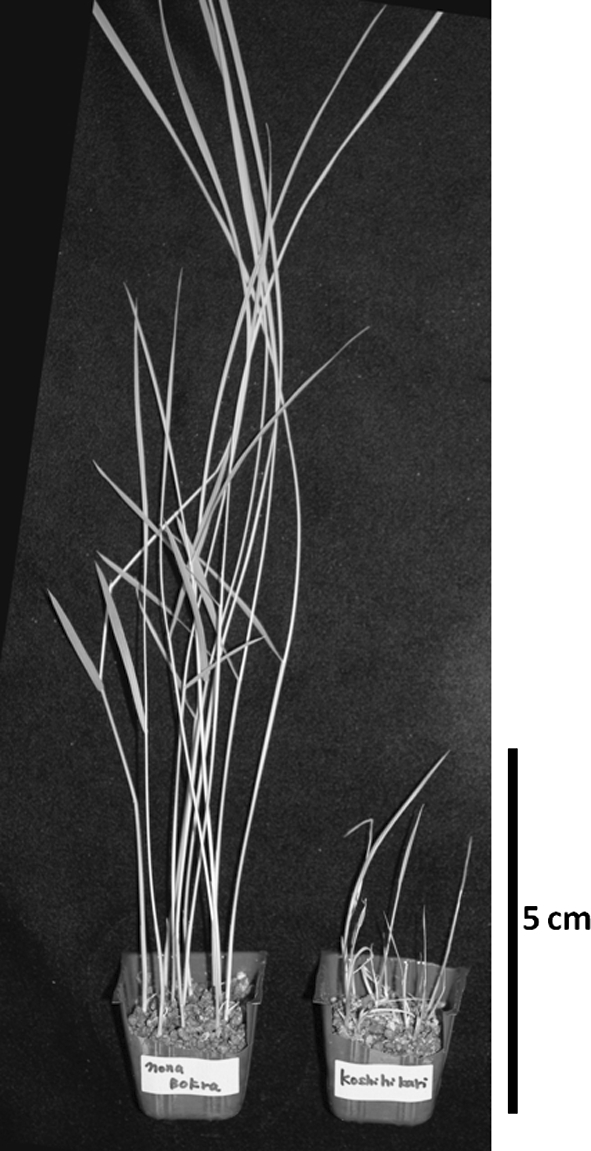
Differences in resistance to bacterial seedling rot between Nona Bokra (*left*) and Koshihikari (*right*)

The BSR ratios of Koshihikari and Nona Bokra were scored 8 days after sowing (Fig. 1). Almost all of the seeds of both cultivars germinated and their shoots emerged from the soil 3–4 days after sowing. The seedlings of Koshihikari showed typical symptoms 6–8 days after sowing: some sheaths showed reddish-brown lesions and most of the others became necrotic. In contrast, most seedlings of Nona Bokra grew normally.

**Fig. 2:**
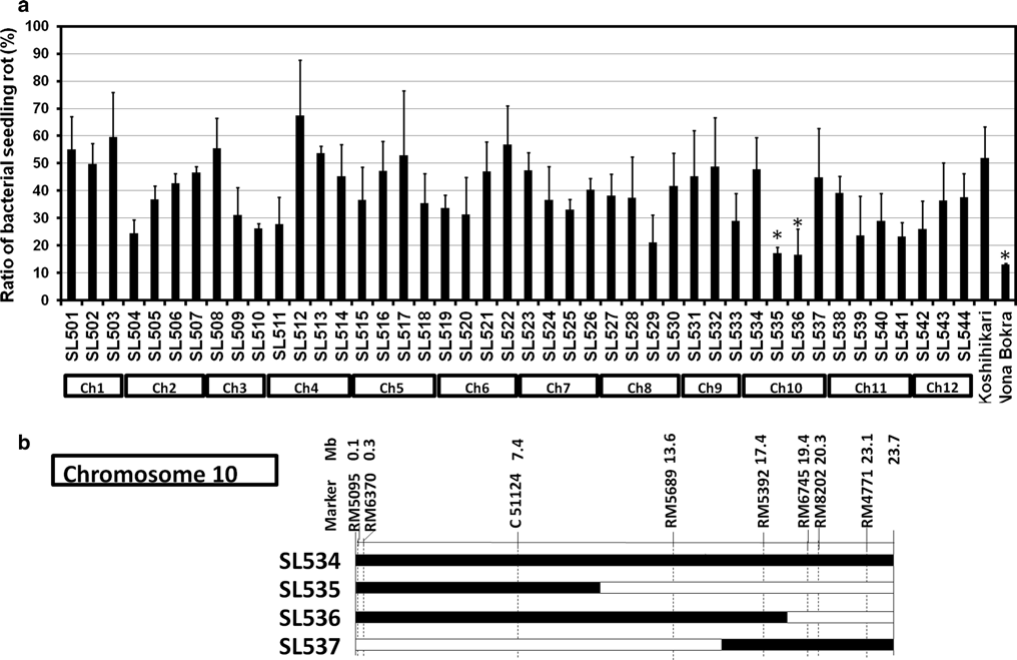
**a** Bacterial seedling rot (BSR) ratios of Koshihikari, Nona Bokra, and 44 CSSLs derived from Koshihikari × Nona Bokra. The BSR ratio of each CSSL was scored 8 days after sowing of inoculated seeds. Chromosome numbers below the *x* axis indicate the main Nona Bokra chromosome segment contained within each CSSL. *Bars* indicate means, and *error bars* indicate SD. *Significant difference from Koshihikari at the 5 % level by Dunnett’s test. **b** Graphical genotypes of chromosome 10 in CSSLs containing substitutions in this chromosome. SSR markers and physical distances based on RAP-DB (IRGSP ver. 1) are indicated above the chromosome maps. *White* and *black* *bars* indicate regions from Koshihikari and Nona Bokra, respectively

To identify the chromosomal segments involved in resistance to BSR, we determined the BSR ratios of the parents and the 44 CSSLs (Fig. 2a). The ratios of Koshihikari and Nona Bokra were 52.0 and 13.0 %, respectively. The BSR ratio varied widely among the CSSLs ranging from 16.6 to 67.4 %. The BSR ratios of 11 CSSLs (SL504, SL510, SL511, SL529, SL533, SL535, SL536, SL539, SL540, SL541, and SL542) were <30.0 %; two of these—SL535 and SL536—had significantly lower ratios (17.1 and 16.6 %, respectively) than the Koshihikari control (*P* < 0.05 by Dunnett’s test). Both had segments of chromosome 10 derived from Nona Bokra (Fig. 2b). Therefore, we hypothesized that this region of chromosome 10 might be involved in the difference in the BSR ratio between Nona Bokra and Koshihikari.

### Detection of a QTL for resistance to BSR

**Fig. 3:**
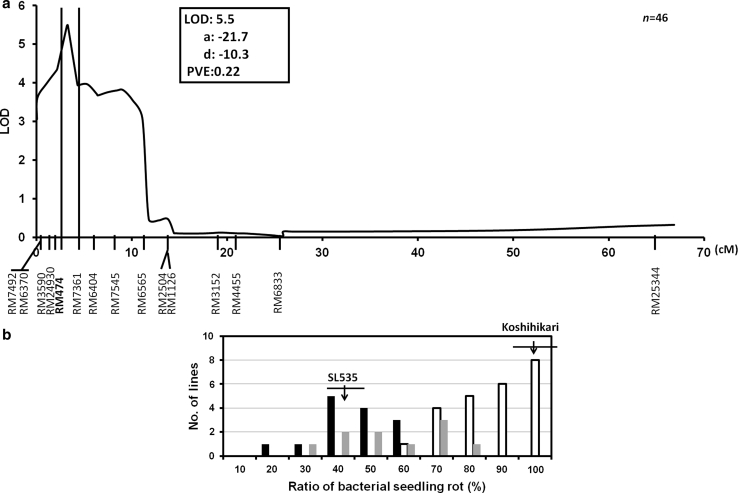
Chromosomal location of a QTL for resistance to bacterial seedling rot (BSR) on the short arm of chromosome 10 and allelic differences for marker RM474. **a** The log-likelihood curve indicates a putative QTL position on chromosome 10 in an F_5_ population derived from Koshihikari × SL535. *LOD* logarithm of odds; *a* additive effect of Nona Bokra allele, *d* dominance effect of Nona Bokra allele, *PVE* percentage of variance explained. **b** Frequency distribution of the BSR ratio in F_5_ plants showing the three genotype classes of SSR marker RM474, which was found to be linked to the QTL. Labels of *x* axis indicate maximum BSR ratio in each bin. Genotypes of RM474 are represented as *white bars* (homozygous for Koshihikari allele), *gray bars* (heterozygous allele), and *black bars* (homozygous for Nona Bokra allele). The BSR ratios in the F_5_ population were determined 8 days after sowing. *Arrows* indicate the mean values for SL535 and Koshihikari; *horizontal lines* across the arrows indicate the standard deviations

To confirm the presence of a putative QTL and to verify the effect of each allele, we developed advanced progeny from a cross between SL535 and Koshihikari. A QTL analysis using an F_5_ population derived from this cross revealed a wide range of variation in the BSR ratio (14.2 to 96.7 %) and detected one QTL between SSR markers RM474 and RM7361 on the short arm of chromosome 10 (Fig. 3a). This QTL accounted for 22 % of the phenotypic variance in the F_5_ plants, and the Nona Bokra allele decreased the BSR ratio by 21.7 % (Fig. 3a). On the basis of the genotype at RM474, the SSR marker most closely linked to the QTL, we classified the F_5_ plants as homozygous for the Nona Bokra allele, homozygous for the Koshihikari allele, or heterozygous (Fig. 3b). F_5_ plants homozygous for the Koshihikari allele showed a high BSR mean ratio (81.4 %) ranging from 53.3 to 96.7 %. In contrast, the BSR mean ratio was 40.7 % ranging from 14.2 to 57.5 % in plants homozygous for the Nona Bokra allele and 50.5 % ranging from 24.2 to 73.3 % in the heterozygous plants. The distribution of BSR ratios of plants homozygous for the Nona Bokra allele was shifted toward lower ratios than the distribution for heterozygotes. These results clearly confirmed the existence of a QTL on the short arm of chromosome 10 and that the Nona Bokra allele at the QTL decreased the BSR ratio.

### Fine mapping of the QTL for resistance to BSR

**Fig. 4:**
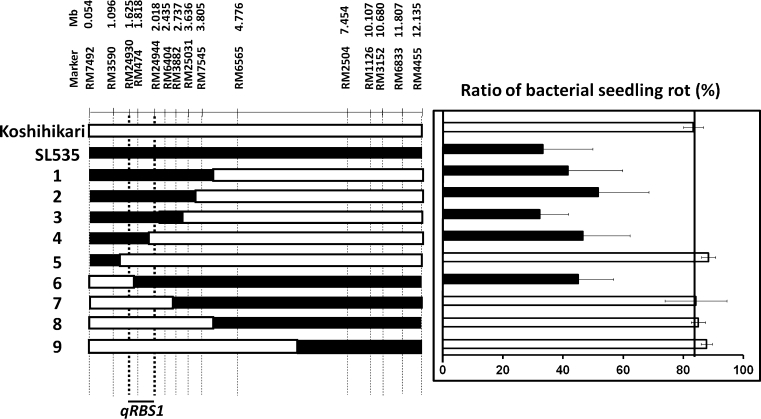
Substitution mapping of a QTL controlling resistance to bacterial seedling rot (BSR) on the short arm of chromosome 10 in a set of nine homozygous F_4_ lines derived from Koshihikari × SL535. (*left*) Graphical genotypes. *Black* denotes regions homozygous for Nona Bokra marker alleles; *white* denotes regions homozygous for Koshihikari marker alleles. The candidate QTL (*qRBS1*) is indicated at the bottom. (*right*) The BSR ratio for each of the nine lines. *Bars* indicate means and *error bars* indicate SD. *Vertical line* indicates mean BSR score of Koshihikari. *Black* indicates a significant difference from Koshihikari at the 5 % level by Dunnett’s test

To further delimit the candidate genomic region of the QTL for BSR, we genotyped 128 F_2_ plants derived from a cross between SL535 and Koshihikari and identified nine homozygous lines with recombination near RM474 (Fig. 4). We checked the genome of the lines and the parental cultivars (Koshihikari and Nona Bokra) by the SNP analysis and found that the remaining genome in the lines was identical to that of Koshihikari except for the target region harboring *qRBS1* (data not shown). Five lines (Nos. 1, 2, 3, 4, and 6) showed a low BSR ratio (32.2 to 51.7 %), whereas four (Nos. 5, 7, 8, and 9) showed a high BSR ratio (84.1 to 88.3 %; Fig. 4). These two phenotypic groups were associated with genotype classes that were homozygous for the Nona Bokra allele and the Koshihikari allele, respectively (Fig. 4). Together, the genotype and phenotype information clearly delimit the QTL for BSR ratio between SSR markers RM24930 and RM24944 (a 393-kb interval in the Nipponbare genome reference sequence) on chromosome 10 (Fig. 4). We have designated this QTL as *qRBS1* (*quantitative trait locus for RESISTANCE TO BACTERIAL SEEDLING ROT 1*), following the nomenclature recommended by McCouch and CGSNL (Committee on Gene Symbolization 2008).

## Discussion

### Identification of a major QTL for BSR in rice

Genetic analysis of BSR resistance is very difficult because the occurrence of BSR is highly affected by environmental conditions (Azegami 2009; Mogi 1984a, b, c). Therefore, only a few cultivars have been reported as partially resistant to BSR on the basis of disease lesion size and bacterial growth in plants (Goto 1983a; Hirashima and Wakimoto 1983; Sayler et al. 2006). Until now, no QTLs for BSR resistance have been reported.

Recent progress in genomics has enhanced understanding of the genetic basis of agronomic traits in rice, including those controlled by multiple loci (Yamamoto et al. 2009; Yano and Sasaki 1997). Advanced backcross progeny such as CSSLs can be useful for genetic analysis (Fukuoka et al. 2010b). Because each CSSL has only one or a few segment substitutions, it is possible to detect QTLs with minor effects generated by the substituted segments (Ebitani et al. 2005; Fukuoka et al. 2010a; Kubo et al. 2002; Marzougui et al. 2011; Takai et al. 2007). We were able to perform reliable assessment of BSR resistance using CSSLs, and we successfully identified a QTL for resistance to BSR, *qRBS1*, located on the short arm of chromosome 10.

Nona Bokra showed a low BSR ratio (13.0 %; Fig. 2a). Although SL535 and SL536, which have segments from chromosome 10 of Nona Bokra, showed lower BSR ratios than Koshihikari, SL534, which contained the same segment of chromosome 10, was not resistant (Fig. 2). Because SL534 also contained a very small segment of chromosome 6 (data not shown), this segment might influence the resistance to BSR. Conversely, several lines other than SL535 and SL536 also showed resistance to BSR, but did not contain chromosome 10 segments from Nona Bokra (Fig. 2a), indicating that *qRBS1* is only one QTL for BSR resistance segregating in this population. This may explain why SL535 is somewhat more susceptible to BSR than Nona Bokra, although it has a significantly lower BSR ratio than Koshihikari.

The conditions for assessment of resistance to BSR used in this experiment were severe. To characterize the effectiveness of *qRBS1* under agricultural conditions, we are currently comparing the resistance to BSR between a near-isogenic line (NIL) containing the Nona Bokra allele of *qRBS1* and Koshihikari, with or without agricultural chemicals and under mild disease conditions.

The candidate genomic region of *qRBS1* was mapped to the interval between RM24930 and RM24944 by substitution mapping (Fig. 4). According to the QTL Annotation Rice Online Database [Q-TARO, http://qtaro.abr.affrc.go.jp/; (Yonemaru et al. 2010)], no QTLs related to disease resistance have been reported in this region. Therefore, *qRBS1* appears to be a novel QTL. *qRBS1* was delimited to a 393-kb region in the Nipponbare genome reference sequence. The Rice Annotation Project database [http://rapdb.dna.affrc.go.jp/ (Ohyanagi et al. 2006)] predicts 47 genes in the candidate region for *qRBS1*. Among the predicted genes, there are three genes similar to NB-ARC domain containing protein and a gene similar to NBS-LRR class disease resistance protein. However, morphological and physiological functions of *qRBS1* are not yet known. Therefore, it is difficult to identify the actual candidate genes for *qRBS1* from among these many predicted genes. Further delimitation of the candidate genomic region will be necessary to identify the gene underlying the QTL.

### Progress toward improvement of resistance to BSR

Recently, some bacteriophages were isolated to lyse some strains of *B. glumae* and suppress BSR and were reported to be more effective than pesticides (Adachi et al. 2012). However, in the actual agricultural phase, various bacteriophages lytic to a wide range of *B. glumae* are needed. Thus, it remains to be necessary to breed a resistant cultivar to BSR.

To utilize *qRBS1* for breeding, we are currently developing an NIL for *qRBS1* in the Koshihikari genetic background. As noted above, some lines which do not have the Nona Bokra allele of *qRBS1* also showed resistance to BSR, suggesting that QTLs other than *qRBS1* are present in Nona Bokra. Thus, it may be necessary to combine *qRBS1* with those QTLs to achieve the level of BSR resistance seen in Nona Bokra. Because we used only one strain to assess the resistance of *qRBS1*, it is still unknown whether *qRBS1* is race specific. To characterize the effectiveness of *qRBS1*, we are planning to assess the resistance of *qRBS1* by several strains collected at a number of areas of Japan. Furthermore, to breed cultivars with stable resistance, it is necessary to search for resistant cultivars other than Nona Bokra to identify additional QTLs different from *qRBS1*.

Seeds contaminated with *B. glumae* are sown and transplanted into fields, and in some cases BSR appears (Azegami 2009; Azegami et al. 1988; Ham et al. 2011a; Tsushima 1996; Tsushima et al. 1989, 1991, 1996). Occasionally, the typical symptoms do not appear and plants with leaf sheaths infected seem to grow normally. However, at heading, panicles are infected by vertical distribution from contaminated leaf sheaths. Plants, which are located near the diseased plants by primary infection, are also attacked by pathogen as secondary infection. The infection reduces yield owing to spikelet abortion, and infected seeds cause BSR and grain rot in the next generation (Ham et al. 2011b). BSR and bacterial grain rot have been widespread in Japan (Goto and Ohata 1956; Goto et al. 1987; Kurita and Tabei 1967; Uematsu et al. 1976a), East Asia (Azegami 2009; Chien and Chang 1987; Cottyn et al. 1996a, b; Jeong et al. 2003; Luo et al. 2007; Trung et al. 1993) and Latin America (Nandakumar et al. 2007b; Zeigler and Alvarez 1989). Therefore, it is necessary to breed cultivars, which are resistant to both BSR and bacterial grain rot.

Although *B. glumae* causes both seedling rot and grain rot, no correlation between the resistance to each was observed (Goto 1983a). We tested Nona Bokra and found that it was not resistant to grain rot (data not shown). In addition, the chromosomal position of *qRBS1* is different from those of QTLs reported to be associated with resistance to grain rot (Mizobuchi et al. 2013; Pinson et al. 2010). Therefore, the factors associated with resistance to seedling rot and grain rot appear to be different. Thus, it will be necessary to combine *qRBS1* and QTLs for resistance to bacterial grain rot to breed cultivars with stable resistance to both diseases.
